# miR‐126‐5p expression in the plasma of patients with sepsis‐induced acute lung injury and its correlation with inflammation and immune function

**DOI:** 10.1111/crj.13646

**Published:** 2023-05-29

**Authors:** Xiaoyong Mao, Yiping Wu, Weifeng Xu

**Affiliations:** ^1^ Department of Blood Transfusion Zhuji People's Hospital Shaoxing Zhejiang China; ^2^ Department of Clinical Laboratory The Second People's Hospital of Zhuji Shaoxing Zhejiang China

**Keywords:** acute lung injury, immune function, inflammation, microRNA‐126‐5p, sepsis

## Abstract

**Objective:**

This work was implemented to elucidate the miR‐126‐5p expression in the plasma of patients with sepsis‐induced acute lung injury (ALI) and its correlation with inflammation and immune function.

**Methods:**

The peripheral blood of patients with sepsis‐induced ALI was obtained, and the levels of inflammatory factors (interleukin‐6 [IL‐6], C‐reactive protein [CRP], and procalcitonin [PCT]) were determined. Meanwhile, T lymphocyte subsets (CD3+, CD4+, and CD8+), and immunoglobulins (IgA, IgM, and IgG) were tested. miR‐126‐5p and TRAF6 mRNA expression in plasma was assessed. Receiver operating characteristic (ROC) curve was performed to assess the diagnostic accuracy of miR‐126‐5p in sepsis without ALI and sepsis with ALI. Correlation between miR‐126‐5p expression and clinical indicators was analyzed. The targets of miR‐126‐5p were predicted using the bioinformatics method, and the direct targets were verified through investigations.

**Results:**

miR‐126‐5p expression in plasma of patients with sepsis‐induced ALI was reduced than that of patients with sepsis without ALI. miR‐126‐5p expression was negatively correlated with IL‐6, CRP, and PCT but positively correlated with IgA, IgM, and IgG as well as CD3+, CD4+, and CD8+ in patients with sepsis‐induced ALI. ROC curve suggested that miR‐126‐5p (AUC: 0.777; 95%CI: 0.689–0.866) could distinguish patients with sepsis with ALI from patients with sepsis without ALI. TRAF6 expression in patients with sepsis‐induced ALI was higher than that in patients with sepsis without ALI. TRAF6 was a target gene of miR‐126‐5p,

**Conclusion:**

This research highlights that miR‐126‐5p is reduced in the plasma of patients with sepsis‐induced ALI, and miR‐126‐5p relates to systemic inflammation and immune function indicators.

## Graphical abstract

The peripheral blood of patients with sepsis‐induced ALI was obtained, and the levels of inflammatory factors (interleukin‐6 [IL‐6], C‐reactive protein [CRP], and procalcitonin [PCT]) were determined. Meanwhile, T lymphocyte subsets (CD3+, CD4+, and CD8+) and immunoglobulins (IgA, IgM, and IgG) were tested. miR‐126‐5p and TRAF6 mRNA expression in plasma was assessed. Receiver operating characteristic (ROC) curve was performed to assess the diagnostic accuracy of miR‐126‐5p in sepsis without ALI and sepsis with ALI. Correlation between miR‐126‐5p expression and clinical indicators was analyzed. The targeting relationship between miR‐126‐5p and TRAF6 was verified.

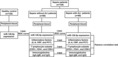

## 1 INTRODUCTION

Sepsis is a complicated and life‐threatening disease that develops from dysregulation of the host's response to infection and leads to organ dysfunction.^1^ The prevention, diagnosis, and treatment of sepsis have become a global health priority, with the developed treatments substantially diminishing organ dysfunction and alleviating the prognosis of patients with sepsis.^2^ Respiratory failure is one of the most significant complications of sepsis, and about half of the patients with severe sepsis may develop into acute lung injury (ALI) or even acute respiratory distress syndrome (ARDS).^3^ Patients with sepsis‐induced ALI have a poor prognosis with a mortality rate of more than 40%.^4^ Therefore, it is urgent to seek for useful therapeutic methods for sepsis‐induced ALI.

Recently, the modulation of microRNA (miRNA) has raised increasing attention in sepsis.^5^, ^6^ miRNAs are non‐coding endogenous RNAs that are implicated in the diagnosis of sepsis via the control of various genes at the post‐transcriptional level through translational repression or mRNA degradation.^7^ Some articles have mentioned that miRNAs could be considered to be a novel biomarker in sepsis.^8^, ^9^ Moreover, there are also studies highlighting that miRNAs could induce or suppress ALI during sepsis.^10^, ^11^ An immune storm leads to organ damage. In addition, sepsis is also a severe infection with organ dysfunction; thus, it is essential to modulate the immune and inflammatory effects in sepsis.^12^ Nowadays, miRNAs have been reported to function in immune modulation.^13^ As one of the miRNAs, miR‐126 could be considered to be a biomarker for the therapy of infection, or autoimmune diseases.^14^ It is also demonstrated that miR‐126‐5p is involved in the regulation of sepsis‐induced liver injury.^15^ miRNA has been indicated to lead to regulatory and protective roles in barrier functions.^16^ Interestingly, miR‐126‐5p enhances the tight junction protein expression, indicating a potent mechanism by which miR‐126 may alleviate lipopolysaccharide (LPS)‐induced lung injury.^11^ It has been reported that nuclear transcription factor‐κB (NF‐κB) specifically binds to the B lymphocyte immunoglobulin kappa light chain gene enhancer B sequence GGGACTTTCC, which was first identified in 1986. To date, NF‐κB has been found to be present in almost all mammalian cells and is involved in a variety of biological processes such as inflammatory response^17^ and immune response^18^ through the regulation of genes encoding acute phase response proteins, cytokines, and immune regulatory molecules. NF‐κB‐mediated signaling pathways are an important pathogenesis of ALI.^19^ It has been shown that miR‐126 is involved in regulating the NF‐κB signaling pathway.^20^, ^21^, ^22^ Evidence has demonstrated that tumor necrosis factor (TNF) receptor‐associated factor 6 (TRAF6) can activate the NF‐κB signaling pathway.^23^, ^24^ Meanwhile, the bioinformatics website StarBase (https://starbase.sysu.edu.cn/) and a previous study have shown that TRAF6 is a target gene of miR‐126.^25^ Therefore, in this study, we speculated that miR‐126 targeted TRAF6 gene to regulate the NF‐κB signaling pathway, which may be associated with inflammatory response and immune response in sepsis‐induced ALI. Nevertheless, miR‐126‐5p expression in the plasma of patients with sepsis‐induced ALI and its relationship with inflammation and immune function remains to be uncovered. Therefore, we investigated the relationship between miR‐126‐5p expression and the levels of inflammatory factors, T lymphocyte subsets, and immunoglobulins in patients with sepsis‐induced ALI and determined the diagnostic value of miR‐126‐5p.

## 2 METHODS

### 2.1 Ethics

This research was ratified by the ethics committee of The Second People's Hospital of Zhuji, and all participants provided informed consent.

### 2.2 Participants

One hundred and twenty patients with sepsis admitted to the intensive care unit (ICU) center of The Second People's Hospital of Zhuji between January 2017 and January 2020 were enrolled as the research subjects, including 68 males and 52 females (age: 60.92 ± 9.16 years old). Inclusion criteria are the following: (1) patients aged ≥18 years; (2) patients conformed to The Third International Consensus Definitions for Sepsis and Septic Shock (Sepsis‐3)^26^ and diagnosed as sepsis before enrollment; (3) patients admitted to ICU within 24 h after onset of symptoms. Exclusion criteria are as follows: (1) patients died within 24 h of admission; (2) patients with other serious illnesses or complications such as cardiothoracic surgery, severe liver or kidney dysfunction, acute coronary syndrome, malignant tumor, or autoimmune disease; (3) patients who are pregnant or lactating women. Among these 120 patients, there were 60 patients with sepsis‐induced ALI, including 36 males and 24 females (age: 61.43 ± 9.46 years old). All of them were in line with the diagnosis of sepsis‐induced ALI^27^: corresponding primary disease; acute onset, dyspnea, or distress symptoms; chest X‐ray showed bilateral lung infiltration, pulmonary artery wedge pressure less than 18 mmHg, and without signs of left atrial hypertension. Additionally, 120 healthy subjects were selected as a control group, including 63 males and 57 females (age: 59.98 ± 9.09 years).

### 2.3 RT‐qPCR

Peripheral blood was harvested (for patients with sepsis, fasting peripheral blood was collected within 24 h of admission; for healthy subjects, fasting peripheral blood was harvested on the enrollment). Total RNA was extracted using TRIzol (Takara Bio, Dalian, China). A Nanodrop2000 device was then utilized for the testing of the RNA concentration. RNA was reverse transcribed into cDNA using the Mir‐X miR First‐Strand Synthesis Kit (Takara Bio) or the PrimeScript RT reagent kit with gDNA Eraser Kit (Takara Bio). Subsequently, RT‐qPCR was performed on an ABI 7500 quantitative PCR instrument with the SYBR Premix Ex Taq kits (Takara Bio). The PCR primers were displayed in Table 1. U6 was utilized as the loading control for miR‐126‐5p. GAPDH was the internal reference of the TRAF6 mRNA. The determination of relative gene levels was realized with the 2^‐ΔΔCT^ method.

**TABLE 1:** Primer sequences for genes utilized in RT‐qPCR assay.

Genes	Primer sequences (5′‐3′)
miR‐126‐5p	F: CATTATTACTTTTGGTACGCG
	R: Universal primers
U6	F: GCTTCGGCAGCACATATACTAAAAT
	R: CGCTTCACGAATTTGCGTGTCAT
TRAF6	F: TTCATAGCTTGAGCGTTATACCCGAC
	R:CAGACTGATCAAGAATTGTAAGGCGTA
GAPDH	F: TGAAGGTCGGAGTCAACGGATTTGGT
	R: CATGTGGGCCATGAGGTCCACCAC

Abbreviations*:* F, forward; GAPDH, glyceraldehyde‐3‐phosphate dehydrogenase; miR‐126‐5p, microRNA‐126‐5p; R, reverse; TRAF6, TNF receptor associated factor 6.

### 2.4 Detection of inflammatory factors and immunological indicators

The levels of interleukin‐6 (IL‐6) in peripheral blood were examined by ELISA Kits (BD Biosciences, San Diego, CA, USA). Procalcitonin (PCT) was detected by sandwich immunoassay utilizing a mini VIDAS automatic fluorescence immunoassay analyzer (Block Scientific, Bohemia, NY, USA) with VIDAS BRAHMS PCT Quantitative Assay Kit (Block Scientific, Bohemia, NY, USA). T lymphocyte subsets (CD3^+^, CD4^+^, and CD8^+^) were tested by Coulter Epics XL flow cytometer (Beckman Coulter, Miami, FL, USA). C‐reactive protein (CRP) and immunoglobulins (IgA, IgM, IgG) were analyzed by rate nephelometry, and the instruments and reagents were provided by Beckman Coulter (Miami, FL, USA).

### 2.5 Dual‐luciferase reporter gene assay

The StarBase (https://starbase.sysu.edu.cn/) bioinformatics software predicted that miR‐126‐5p had a complementary binding site to the 3′UTR region of TRAF6 gene. To further verify the targeting relationship between miR‐126‐5p and TRAF6, sequences containing the miR‐126‐5p binding site on the TRAF6 3′UTR were amplified and cloned into the pGL3‐basic luciferase plasmids (TaKaRa, Japan) to construct wild type TRAF6 (TRAF6‐WT) recombinant plasmids. The miR‐126‐5p binding site on TRAF6‐WT was mutated utilizing a point mutation kit (TaKaRa) to construct mutant TRAF6 (TRAF6‐MUT) recombinant plasmids (plasmids were designed and constructed by TaKaRa). The 293T cells (National Collection of Authenticated Cell Cultures) were cultured and inoculated in 24‐well plates, and when the cells reached 85% confluence, TRAF6‐WT or TRAF6‐MUT reporter plasmids were cotransfected with miR‐126‐5p mimic and mimic NC into 293T cells according to the Lipofectamine 2000 reagent (Invitrogen, USA) procedures. After 48 h, the luciferase activity of the TRAF6‐WT or TRAF6‐MUT reporter plasmids was measured by implementing the Dual‐Luciferase® Reporter Assay System (Promega, USA). Dual‐luciferase reporter assay was performed with three biological replicates, each with three technical replicates.

### 2.6 Statistics

Data were analyzed utilizing SPSS 21.0 software and GraphPad Prism 6.0 software. The measurement data, in the form of mean ± standard deviation, were analyzed by the t‐test or variance of analysis (ANOVA) with Tukey's pairwise post‐hoc test. The enumeration data, expressed as numbers (percentage), were compared by the chi‐square test. The correlation was analyzed by the Pearson correlation test. Receiver operating characteristic (ROC) curve analysis was performed and the diagnostic accuracy of miR‐126‐5p for the area under the curve (AUC) was estimated. Statistical significance was accepted at *p* < 0.05.

## 3 RESULTS

### 3.1 General characteristics of patients with sepsis

Among the 120 patients with sepsis, there were 60 patients with sepsis without ALI (Sepsis, no ALI) and 60 patients with sepsis without ALI (Sepsis and ALI). No significant differences were witnessed in age, gender, body mass index (BMI), chronic kidney failure, and cardiomyopathy, as well as an infected site between the aforesaid two groups (all *p* > 0.05). However, obvious differences were observed in smoking history, ALI/ARDS score, and FiO2 (%) between the two groups (both *p* < 0.05; Table 2).

**TABLE 2:** General characteristics of patients with sepsis.

Variable	Sepsis, no ALI	Sepsis and ALI	*p* value
	(n = 60)	(n = 60)	
Age (years)	60.42 ± 8.93	61.43 ± 9.46	0.549
Gender			0.581
Male	32 (53.33%)	36 (60.00%)	
Female	28 (46.67%)	24 (40.00%)	
BMI (kg/m^2^)	22.87 ± 3.11	23.38 ± 3.60	0.408
Smoking, No. (%)	20 (33.33%)	36 (60.00%)	0.006
Cardiomyopathy, No. (%)	23 (38.33%)	25 (41.67%)	0.852
Chronic kidney failure, No. (%)	8 (13.33%)	12 (20.00%)	0.463
Infected site			0.909
Lung	19 (31.67%)	17 (28.33%)	
Urinary tract	8 (13.33%)	9 (15.00%)	
Abdominal cavity	26 (43.33%)	24 (40.00%)	
Blood flow	2 (3.33%)	4 (6.67%)	
Skin soft tissue	5 (8.34%)	6 (10.00%)	
ALI/ARDS score	1.2 ± 0.2	3.1 ± 0.7	<0.001
FiO_2_ (%)	34.3 ± 6.7	42.8 ± 8.1	<0.001

Abbreviations: ALI, acute lung injury; ARDS, acute respiratory distress syndrome; BMI, body mass index.

### 3.2 Inflammatory factors in patients with sepsis‐induced ALI

To address the expression of inflammatory factors in sepsis‐induced ALI, we tested the levels of CRP, PCT, and IL‐6 in patients with sepsis without ALI and patients with ALI. There were high levels of the above‐mentioned factors in sepsis‐induced ALI in comparison with those without ALI (all *p* < 0.05; Table 2).

### 3.3 Comparison of immune‐related indicators in patients with sepsis‐induced ALI

In order to observe the levels of immune‐associated indicators in patients with sepsis‐induced ALI, we tested the levels of immunoglobulins (IgA, IgM, and IgG) and T lymphocyte subsets (CD3+, CD4+, and CD8+) The results in Table 3 demonstrated that the levels of immunoglobulins and T lymphocyte subsets in sepsis‐induced ALI were lower than those without ALI (all *p* < 0.05).

**TABLE 3:** Comparison of inflammatory factors and immune‐related indices.

Factors	Sepsis, no ALI	Sepsis and ALI	*p* value
	(n = 60)	(n = 60)	
CRP (mg/L)	151.28 ± 26.93	167.73 ± 31.45	0.003
PCT (ng/ml)	2.71 ± 0.41	3.83 ± 0.49	<0.001
IL‐6 (pg/ml)	253.66 ± 34.41	286.61 ± 42.54	<0.001
IgA (mg/dl)	3.00 ± 0.52	2.55 ± 0.78	<0.001
IgM (mg/dl)	2.08 ± 0.32	1.68 ± 0.26	<0.001
IgG (mg/dl)	1143.08 ± 27.49	1026.47 ± 24.16	<0.001
CD3^+^	64.32 ± 11.29	58.42 ± 10.66	0.004
CD4^+^	43.95 ± 11.03	37.03 ± 9.97	<0.001
CD8^+^	26.52 ± 8.63	24.10 ± 7.55	0.105

*Note*: CRP normal reference value <8 mg/L; PCT normal reference value <0.05 ng/ml; IL‐6 normal reference value < 7 pg/ml.

Abbreviations: ALI, acute lung injury; CRP, C‐reactive protein; PCT, procalcitonin.

### 3.4 miR‐126‐5p expression in patients with sepsis‐induced ALI

**FIGURE 1:**
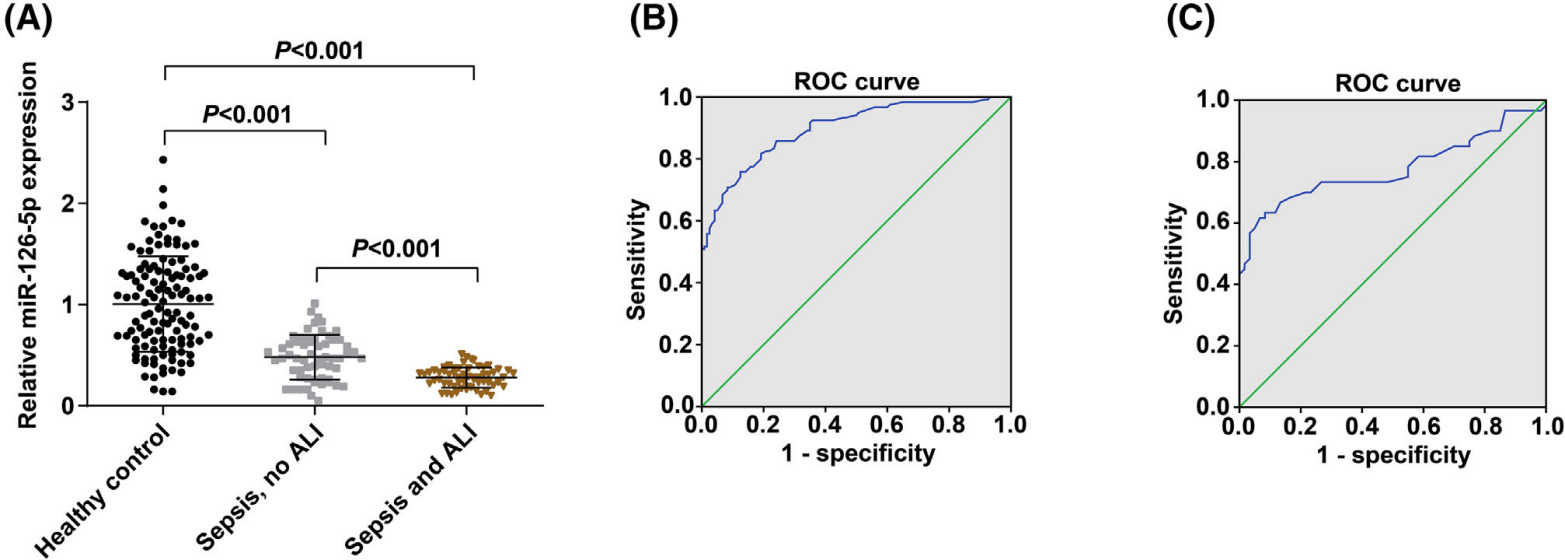
Comparison of miR‐126‐5p expression in patients with sepsis‐induced ALI from those patients with sepsis without ALI. (A). Comparison of miR‐126‐5p expression between patients with sepsis and healthy subjects. (B). Correlation between miR‐126‐5p and sepsis risk by ROC curve. (C). Predictive value of miR‐126‐5p on ALI risk by ROC curve. Abbreviations: ALI, acute lung injury; ROC, receiver operating characteristic.

Plasma miR‐126‐5p expression was diminished in patients with sepsis in comparison with healthy people (*p* < 0.05; Figure 1A). Besides, the ROC curve indicated that (Figure 1B) miR‐126‐5p could well distinguish patients with sepsis from healthy people (AUC: 0.897; 95% CI: 0.859–0.936; *p* < 0.001) with miR‐126‐5p expression at the best cut‐off point of 0.635, (sensitivity: 75.8%; specificity: 87.5%).

Next, plasma miR‐126‐5p expression was reduced in sepsis‐induced ALI in contrast to those without ALI (*p* < 0.05; Figure 1A). Additionally, the ROC curve unveiled that (Figure 1C) miR‐126‐5p could distinguish patients with sepsis‐induced ALI from those patients with sepsis without ALI (AUC: 0.777; 95% CI: 0.689–0.866; *p* < 0.001) with miR‐126‐5p expression at the best cut‐off point of 0.405 (sensitivity: 63.3%; specificity: 91.7%).

### 3.5 Correlation between miR‐126‐5p and clinical experimental parameters

The results in Table 4 unveiled that miR‐126‐5p expression was closely associated with the expression levels of markers of systemic inflammation and bacterial infection such as CRP concentration (r = −0.379, *p* = 0.003), PCT (r = −0.380, *p* = 0.003), and IL‐6 (r = −0.511, *p* < 0.001) in patients with sepsis‐induced ALI. Also, we witnessed that plasma miR‐126‐5p concentration was correlated with immunoglobulin IgA (r = 0.354, *p* = 0.006), IgM (r = 0.371, *p* = 0.004), IgG (r = 0.421, *p* = 0.001), CD3^+^ (r = 0.404, *p* = 0.001), and CD4^+^ (r = 0.310, *p* = 0.016).

**TABLE 4:** Correlation between serum miR‐126‐5p concentration and laboratory markers in patients with sepsis‐induced lung injury.

	Sepsis‐induced lung injury	
	r	*p*
Inflammatory biomarkers		
CRP	−0.379	0.003
PCT	−0.380	0.003
IL‐6	−0.511	<0.001
Immune‐related indices		
IgA	0.354	0.006
IgM	0.371	0.004
IgG	0.421	0.001
CD3^+^	0.404	0.001
CD4^+^	0.310	0.016
CD8^+^	0.125	ns

Abbreviations: CRP, C‐reactive protein; IL‐6, interleukin‐6; PCT, procalcitonin.

### 3.6 Genetic validation assay for miR‐126‐5p and its target gene TRAF6

**FIGURE 2:**
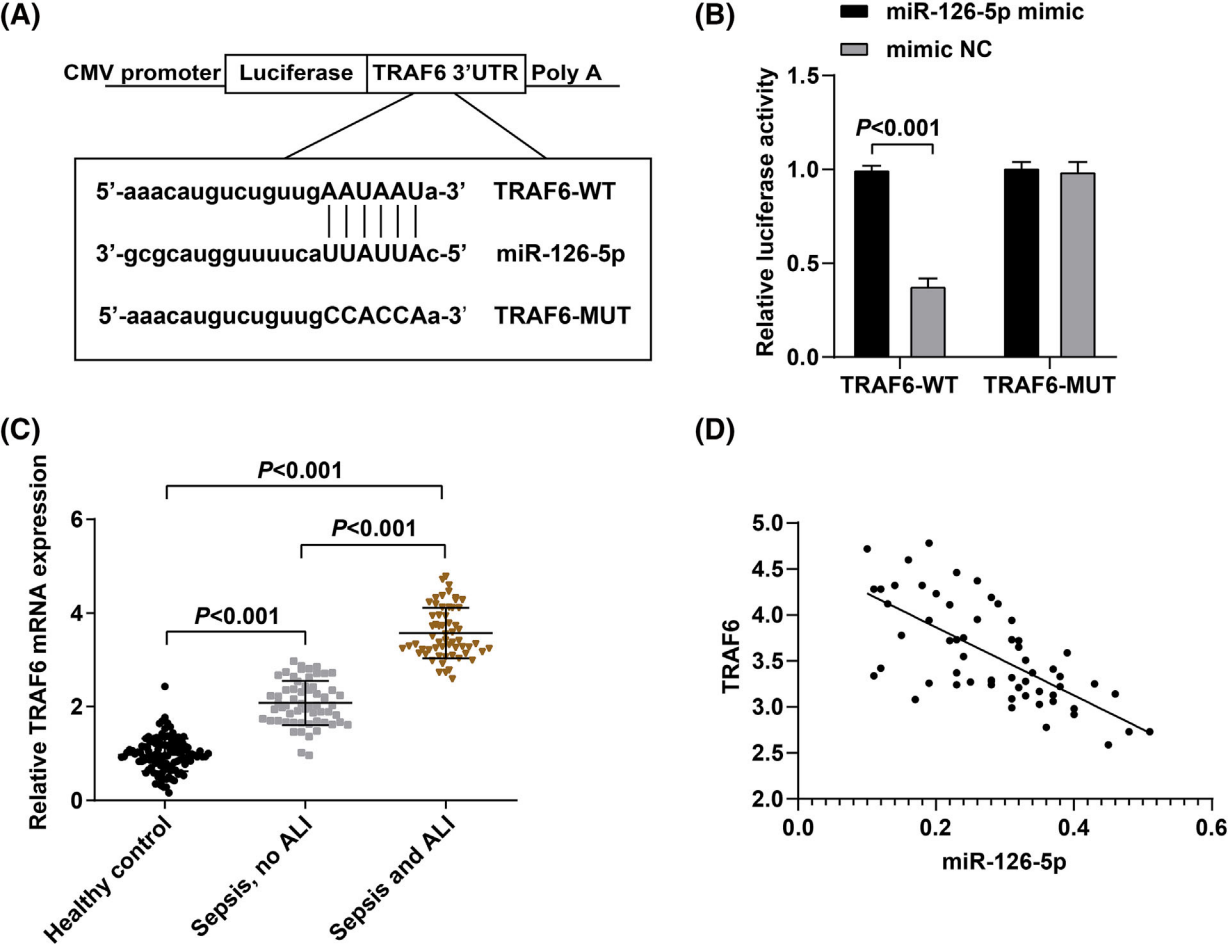
TRAF6 is a target gene of miR‐126‐5p. (A). The binding site between miR‐126‐5p and TRAF6; (B). The results of luciferase reporter gene assay; (C). TRAF6 expression in patients with sepsis‐induced ALI; (D). Correlation analysis of miR‐126‐5p and TRAF6 expression in patients with sepsis‐induced ALI. Abbreviation: ALI, acute lung injury.

According to the prediction analysis, miR‐126‐5p had a binding site with TRAF6 3′UTR (Figure 2A). To verify whether miR‐126‐5p inhibited TRAF6 expression by binding to the 3′UTR of TRAF6, the dual‐luciferase reporter gene assay was performed. TRAF6‐WT or TRAF6‐MUT luciferase reporter was transfected into 293T cells together with miR‐126‐5p mimic or mimic NC. Overexpression of miR‐126‐5p repressed the luciferase activity of the TRAF6‐WT reporter plasmids, but did not affect the luciferase activity of the TRAF6‐MUT reporter plasmids (Figure 2B). Thus, these results suggested that TRAF6 was a direct target of miR‐126‐5p. We further examined TRAF6 expression in patients with sepsis‐induced ALI utilizing RT‐qPCR and the findings suggested (Figure 2C) that plasma TRAF6 expression levels in patients with sepsis‐induced ALI were elevated compared with those in patients with sepsis without ALI (*p* < 0.05). Further analysis of the correlation between TRAF6 and miR‐126‐5p in patients with sepsis‐induced ALI unraveled that miR‐126‐5p was negatively correlated with TRAF6 in patients with sepsis‐induced ALI.

## 4 DISCUSSION

ALI is a primary complication in sepsis, and miRNAs play vital parts in sepsis‐associated disorders.^28^ The biology of sepsis‐induced ALI is multifactorial, which involves the secretion of inflammatory factors along with the damage to the lung microvascular barrier.^29^ As reported, miRNAs participate in diverse physiological and pathological processes, including lung injury.^30^ In this research, we aimed at finding out a novel method for sepsis‐induced ALI by evaluating the expression of miR‐126‐5p in the plasma of patients with sepsis‐induced ALI and its relationship with inflammation and immune function.

Emerging works have highlighted that miRNAs are of significance in regulating oxidative stress and inflammation, and are linked to sepsis‐induced ALI. For instance, Jiang et al. have pointed out that miR‐155 accelerates macrophage proliferation and aggregates septic lung injury.^31^ Wang et al. have supported that miR‐326, as an autoimmunity‐associated miRNA, could provide a latent molecular basis in sepsis‐induced ALI.^32^ In our research, we observed a reduced miR‐126‐5p in the plasma of patients with sepsis and sepsis‐induced ALI. Complimentary to this, author X et al, have reported decreased miR‐126 expression in sepsis and that reduced serum levels of miR‐126 are associated with sepsis severity.^33^ Another research has mentioned that miR‐126‐5p is downregulated in LPS‐induced ALI mice, and restoration of miR‐126‐5p may alleviate ALI by decreasing its downstream gene VEGFA.^30^ As stated above, miR‐126 might be downregulated in either sepsis or LPS‐induced ALI, and our work highlighted that miR‐126 expression was lower in patients with sepsis with ALI than that in patients with sepsis without ALI. Besides, most previous articles tested miR‐126‐5p expression in serum, while this paper tested it in plasma, which is a highlight of this paper because the results of plasma detection will be more accurate.

Some proinflammatory cytokines are implicated in the pathogenesis of sepsis.^34^ As previously described, pathogens activate the secretion of inflammatory factors, contributing to sepsis‐induced ALI or ARDS.^35^ In addition, it is suggested that reducing these cytokines has some protective functions in animal models with acute fulminant infections.^36^ In our article, we found that the levels of pro‐inflammatory factors were enhanced in sepsis‐induced ALI. Meanwhile, plasma miR‐126‐5p concentration correlated to systemic inflammation in sepsis‐induced ALI. In recent years, it is reported that the sepsis‐associated severe infection originates from an imbalance between anti‐inflammatory and/or inflammatory factors.^37^ Meanwhile, miR‐126 impairs sepsis inflammation and improves the prognosis of sepsis.^38^ Nong et al. in their study have stated that the downregulation of miR‐126 suppresses the levels of proinflammatory factors and elevates anti‐inflammatory factor levels in septic rats, which could relieve persistent damage resulting from excessive inflammation.^21^ A previous study has reported that plasma miR‐126 levels are elevated in smokers who had completely quit smoking.^39^ In this study, more smokers were found in patients with sepsis with ALI than in patients with sepsis without ALI, and miR‐126‐5p expression was lower in patients with sepsis with ALI than that in patients with sepsis without ALI, indicating that miR‐126‐5p expression may be associated with smoking history. Furthermore, we investigated the relationship between miR‐126 expression and the levels of inflammatory factors, T lymphocyte subsets, and immunoglobulins in patients with sepsis‐induced ALI and determined the diagnostic value of miR‐126‐5p. The focus of the research was aimed at patients with sepsis‐induced ALI. Therefore, we did not analyze the correlation among miR‐126‐5p expression and the levels of inflammatory factors, T lymphocyte subsets, and immunoglobulins in patients with sepsis without ALI, which is similar to other scholars' research.^40^

In addition, we also found that the levels of immunoglobulins and T lymphocyte subsets were decreased in patients with sepsis‐induced ALI. Meanwhile, miR‐126‐5p expression was found to correlate with markers of systemic inflammation and immunological function indicators in patients with sepsis‐induced ALI. Similar to our findings, evidence has shown that the reduced plasma levels of endogenous immunoglobulins have a correlation to decreased survival in severe sepsis.^41^ Acquired immunosuppression is an important event in sepsis, leading to the hypothesis that stimulating the immune response and/or replacing the components of the individual immune system could be a promising therapeutic approach.^42^ T lymphocytes are vital cells in the immune system, both serving as the cell‐modulated immune effector cells.^43^ As reported, overexpression of miR‐126 is capable of regulating the immune response (Th1/Th17), and exerting functions in immune signal transduction and immune cell migration.^44^ Additionally, miR‐126 impedes the development of the immune response and modulates the differentiation of T lymphocytes with the involvement of Th2 or Tregs.^14^ Furthermore, in our paper, ROC analysis showed the miR‐126‐5p expression for the evaluation of the AUC of sepsis: 0.897; for sepsis‐induced ALI: 0.777. These findings reveal that miR‐126‐5p might be a potential diagnostic agent for sepsis‐induced ALI. As previously reported, TRAF6 expression is up‐regulated in LPS‐induced ALI.^45^ Bioinformatics website along with a related study has revealed that TRAF6 is a target gene of miR‐126.^25^ In this study, miR‐126‐5p was reduced in sepsis‐induced ALI, and it was speculated that the target gene TRAF6 would be elevated in sepsis‐induced ALI. We have tested the expression of TRAF6 in patients with sepsis‐induced ALI, and the results were as speculated: TRAF6 expression was elevated in sepsis‐induced ALI.

In conclusion, this research provides evidence that miR‐126‐5p is decreased in sepsis‐induced ALI, and miR‐126‐5p correlates to systemic inflammation and immune function indicators. This emphasizes that miR‐126‐5p might be a potential biomarker for the diagnosis of sepsis‐induced ALI. In this study, we did not detect the roles of other miRNAs in sepsis‐induced ALI, which could be a limitation of this study, and we would further investigate this aspect in future research.

## AUTHOR CONTRIBUTIONS

Weifeng Xu and Xiaoyong Mao finished study design, Xiaoyong Mao and Yiping Wu finished experimental studies, Yiping Wu and Weifeng Xu finished data analysis, Xiaoyong Mao and Weifeng Xu finished manuscript editing. All authors read and approved the final manuscript.

## CONFLICT OF INTEREST STATEMENT

The authors have no conflicts of interest to declare that are relevant to the content of this article.

## ETHICAL STATEMENT

This research was approved by the ethics committee of The Second People's Hospital of Zhuji, and all participants provided informed consent.

## Data Availability

The original contributions presented in the study are included in the article/Supplementary Material, further inquiries can be directed to the corresponding author.

## References

[1] Cecconi M , Evans L , Levy M , Rhodes A . Sepsis and septic shock. Lancet. 2018;392(10141):75‐87. doi:10.1016/S0140-6736(18)30696-2 29937192

[2] Armstrong BA , Betzold RD , May AK . Sepsis and septic shock strategies. Surg Clin North am. 2017;97(6):1339‐1379. doi:10.1016/j.suc.2017.07.003 29132513

[3] Zhou X , Liao Y . Gut‐lung crosstalk in sepsis‐induced acute lung injury. Front Microbiol. 2021;12:779620. doi:10.3389/fmicb.2021.779620 35003009 PMC8733643

[4] Aziz M , Ode Y , Zhou M , et al. B‐1a cells protect mice from sepsis‐induced acute lung injury. Mol Med. 2018;24(1):26. doi:10.1186/s10020-018-0029-2 30134811 PMC6016888

[5] Wang G , Lin F , Wan Q , Wu J , Luo M . Mechanisms of action of metformin and its regulatory effect on microRNAs related to angiogenesis. Pharmacol Res. 2021;164:105390. doi:10.1016/j.phrs.2020.105390 33352227

[6] Kikuchi A , Naruse A , Sawamura T , Nonaka K . Evaluation of the efficacy of various reagents in improving microRNA extraction. Ann Clin Biochem. 2019;56(3):375‐380. doi:10.1177/0004563219828405 30813744

[7] Kingsley SMK , Bhat BV . Role of microRNAs in sepsis. Inflamm Res. 2017;66(7):553‐569. doi:10.1007/s00011-017-1031-9 28258291

[8] Li J , Xu H , Li N , du W , Ti J , Chen J . Long non‐coding RNA growth arrest specific 5 is downregulated in sepsis‐ALI and inhibits apoptosis by up‐regulating miR‐146a. Bioengineered. 2022;13(2):4146‐4152. doi:10.1080/21655979.2021.2014619 35112981 PMC8974100

[9] Lu Q , Zhang D , liu H , Xu H . miR‐942‐5p prevents sepsis‐induced acute lung injury via targeting TRIM37. Int J Exp Pathol. 2021;102(4‐5):192‐199. doi:10.1111/iep.12413 34716956 PMC8576636

[10] Ling Y , Li ZZ , Zhang JF , et al. MicroRNA‐494 inhibition alleviates acute lung injury through Nrf2 signaling pathway via NQO1 in sepsis‐associated acute respiratory distress syndrome. Life Sci. 2018;210:1‐8. doi:10.1016/j.lfs.2018.08.037 30121199 PMC9673760

[11] Zhou Y , Li P , Goodwin AJ , et al. Exosomes from endothelial progenitor cells improve outcomes of the lipopolysaccharide‐induced acute lung injury. Crit Care. 2019;23(1):44. doi:10.1186/s13054-019-2339-3 30760290 PMC6373158

[12] Hotchkiss RS , Monneret G , Payen D . Sepsis‐induced immunosuppression: from cellular dysfunctions to immunotherapy. Nat Rev Immunol. 2013;13(12):862‐874. doi:10.1038/nri3552 24232462 PMC4077177

[13] Contreras J , Rao DS . MicroRNAs in inflammation and immune responses. Leukemia. 2012;26(3):404‐413. doi:10.1038/leu.2011.356 22182919

[14] Bai Y , Lu W , Han N , Bian H , Zhu M . Functions of miR126 and innate immune response. Yi Chuan. 2014;36(7):631‐636. doi:10.3724/SP.J.1005.2014.0631 25076026

[15] Li Y , Song J , Xie Z , Liu M , Sun K . Long noncoding RNA colorectal neoplasia differentially expressed alleviates sepsis‐induced liver injury via regulating miR‐126‐5p. IUBMB Life. 2020;72(3):440‐451. doi:10.1002/iub.2230 32031750

[16] Zhang ZS , Liu YY , He SS , et al. Pericytes protect rats and mice from sepsis‐induced injuries by maintaining vascular reactivity and barrier function: implication of miRNAs and microvesicles. Mil Med Res. 2023;10(1):13. doi:10.1186/s40779-023-00442-2 36907884 PMC10010010

[17] Jiang X , Chen L , Zhang Z , Sun Y , Wang X , Wei J . Protective and therapeutic effects of Engeletin on LPS‐induced acute lung injury. Inflammation. 2018;41(4):1259‐1265. doi:10.1007/s10753-018-0773-z 29704150

[18] Vallabhapurapu S , Karin M . Regulation and function of NF‐kappaB transcription factors in the immune system. Annu Rev Immunol. 2009;27(1):693‐733. doi:10.1146/annurev.immunol.021908.132641 19302050

[19] Zhang B , Liu ZY , Li YY , et al. Antiinflammatory effects of matrine in LPS‐induced acute lung injury in mice. Eur J Pharm Sci. 2011;44(5):573‐579. doi:10.1016/j.ejps.2011.09.020 22019524

[20] Feng X , Tan W , Cheng S , et al. Upregulation of microRNA‐126 in hepatic stellate cells may affect pathogenesis of liver fibrosis through the NF‐kappaB pathway. DNA Cell Biol. 2015;34(7):470‐480. doi:10.1089/dna.2014.2760 25974152

[21] Nong A , Li Q , Huang Z , et al. MicroRNA miR‐126 attenuates brain injury in septic rats via NF‐kappaB signaling pathway. Bioengineered. 2021;12(1):2639‐2648. doi:10.1080/21655979.2021.1937905 34115555 PMC8806573

[22] Liu W , Song J , Feng X , Yang H , Zhong W . LncRNA XIST is involved in rheumatoid arthritis fibroblast‐like synoviocytes by sponging miR‐126‐3p via the NF‐kappaB pathway. Autoimmunity. 2021;54(6):326‐335. doi:10.1080/08916934.2021.1937608 34165008

[23] Rajagopalan S , Lee EC , DuPrie ML , Long EO . TNFR‐associated factor 6 and TGF‐beta‐activated kinase 1 control signals for a senescence response by an endosomal NK cell receptor. J Immunol. 2014;192(2):714‐721. doi:10.4049/jimmunol.1302384 24337384 PMC4556140

[24] Park JE , Kim YI , Yi AK . Protein kinase D1 is essential for MyD88‐dependent TLR signaling pathway. J Immunol. 2009;182(10):6316‐6327. doi:10.4049/jimmunol.0804239 19414785 PMC2683622

[25] Wu Y , Song LT , Li JS , Zhu DW , Jiang SY , Deng JY . MicroRNA‐126 regulates inflammatory cytokine secretion in human gingival fibroblasts under high glucose via targeting tumor necrosis factor receptor associated factor 6. J Periodontol. 2017;88(11):e179‐e187. doi:10.1902/jop.2017.170091 28598282

[26] Singer M , Deutschman CS , Seymour CW , et al. The third international consensus definitions for sepsis and septic shock (Sepsis‐3). Jama. 2016;315(8):801‐810. doi:10.1001/jama.2016.0287 26903338 PMC4968574

[27] Force ADT , Ranieri VM , Rubenfeld GD , et al. Acute respiratory distress syndrome: the Berlin definition. Jama. 2012;307(23):2526‐2533. 22797452 10.1001/jama.2012.5669

[28] Rogobete AF , Sandesc D , Bedreag OH , et al. MicroRNA expression is associated with sepsis disorders in critically ill polytrauma patients. Cell. 2018;7(12):271. doi:10.3390/cells7120271 PMC6316368 30551680

[29] Huppert LA , Matthay MA , Ware LB . Pathogenesis of acute respiratory distress syndrome. Semin Respir Crit Care Med. 2019;40(1):31‐39. doi:10.1055/s-0039-1683996 31060086 PMC7060969

[30] Tang R , Pei L , Bai T , Wang J . Down‐regulation of microRNA‐126‐5p contributes to overexpression of VEGFA in lipopolysaccharide‐induced acute lung injury. Biotechnol Lett. 2016;38(8):1277‐1284. doi:10.1007/s10529-016-2107-2 27146208

[31] Jiang K , Yang J , Guo S , Zhao G , Wu H , Deng G . Peripheral circulating exosome‐mediated delivery of miR‐155 as a novel mechanism for acute lung inflammation. Mol Ther. 2019;27(10):1758‐1771. doi:10.1016/j.ymthe.2019.07.003 31405809 PMC6822235

[32] Wang Z , Yan J , Yang F , Wang D , Lu Y , Liu L . MicroRNA‐326 prevents sepsis‐induced acute lung injury via targeting TLR4. Free Radic Res. 2020;54(6):408‐418. doi:10.1080/10715762.2020.1781847 32530324

[33] Chen C , Zhang L , Huang H , et al. Serum miR‐126‐3p level is down‐regulated in sepsis patients. Int J Clin Exp Pathol. 2018;11(5):2605‐2612. 31938374 PMC6958305

[34] Kumar S , Ingle H , Prasad DVR , Kumar H . Recognition of bacterial infection by innate immune sensors. Crit Rev Microbiol. 2013;39(3):229‐246. doi:10.3109/1040841X.2012.706249 22866947

[35] Luyt CE , Bouadma L , Morris AC , et al. Pulmonary infections complicating ARDS. Intensive Care Med. 2020;46(12):2168‐2183. doi:10.1007/s00134-020-06292-z 33175277 PMC7656898

[36] Wiersinga WJ , Leopold SJ , Cranendonk DR , van der Poll T . Host innate immune responses to sepsis. Virulence. 2014;5(1):36‐44. doi:10.4161/viru.25436 23774844 PMC3916381

[37] Chepurnova DA , Samoilova EV , Anisimov AA , Verin AD , Korotaeva AA . Compounds of IL‐6 receptor complex during acute lung injury. Bull Exp Biol Med. 2018;164(5):609‐611. doi:10.1007/s10517-018-4042-9 29577202 PMC6428418

[38] Wang HF , Wang YQ , Dou L , et al. Influences of up‐regulation of miR‐126 on septic inflammation and prognosis through AKT/Rac1 signaling pathway. Eur Rev Med Pharmacol Sci. 2019;23(5):2132‐2138. doi:10.26355/eurrev_201903_17257 30915758

[39] Fujii S , Sugiura T , Dohi Y , Ohte N . MicroRNA in atherothromobosis: is it useful as a disease marker? Thromb J. 2016;14(Suppl 1):21. doi:10.1186/s12959-016-0112-2 27766047 PMC5056487

[40] Wang ZF , Yang YM , Fan H . Diagnostic value of miR‐155 for acute lung injury/acute respiratory distress syndrome in patients with sepsis. J Int Med Res. 2020;48(7):300060520943070. doi:10.1177/0300060520943070 32720541 PMC7388133

[41] Ando Y , Inoue S , Kawashima T , Okashiro M , Kotani J , Nishiyama T . Intravenous immunoglobulin G modulates the expression of sepsis‐induced coagulopathy factors and increases serum IgM levels: a prospective, single‐center intervention study. Kobe J Med Sci. 2020;66(1):E32‐E39. 32814755 PMC7447101

[42] Jarczak D , Kluge S , Nierhaus A . Use of intravenous immunoglobulins in sepsis therapy‐a clinical view. Int J Mol Sci. 2020;21(15):5543. doi:10.3390/ijms21155543 32756325 PMC7432410

[43] Yang G , Hu RY , Deng AJ , Huang Y , Li J . Effects of electro‐acupuncture at Zusanli, Guanyuan for sepsis patients and its mechanism through immune regulation. Chin J Integr Med. 2016;22(3):219‐224. doi:10.1007/s11655-016-2462-9 26825083

[44] Pandey RK , Sundar S , Prajapati VK . Differential expression of miRNA regulates T cell differentiation and plasticity during visceral Leishmaniasis infection. Front Microbiol. 2016;7:206. 26941729 10.3389/fmicb.2016.00206 PMC4766295

[45] Gao W , Zhang Y . Depression of lncRNA MINCR antagonizes LPS‐evoked acute injury and inflammatory response via miR‐146b‐5p and the TRAF6‐NFkB signaling. Mol Med. 2021;27(1):124. doi:10.1186/s10020-021-00367-3 34602057 PMC8489090

